# Changes in PH in exhaled breath condensate after specific bronchial challenge test in patients with chronic hypersensitivity pneumonitis: a prospective study

**DOI:** 10.1186/s12890-015-0100-5

**Published:** 2015-09-30

**Authors:** I. Ojanguren, M J Cruz, A. Villar, M. Sanchez-Ortiz, F. Morell, X. Munoz

**Affiliations:** Servicio de Neumología, Departamento de Medicina, Hospital Universitario Vall d´Hebron, Universidad Autónoma de Barcelona, Catalonia, Spain; CIBER Enfermedades Respiratorias (Ciberes), Barcelona, Spain; Departamento de Biología Celular, Fisiología e Inmunología, Universidad Autónoma de Barcelona, Catalonia, Spain; Servei de Pneumologia, Hospital Universitari Vall d’Hebron, Passeig Vall d’Hebron, 119, 08035 Barcelona, Spain

**Keywords:** Inflammation, Pulmonary fibrosis, Environmental exposure

## Abstract

**Background:**

The aim of this study was to investigate the influence of the specific inhalation challenge (SIC) on changes of pH values in exhaled breath condensate (EBC) in patients with hypersensitivity pneumonitis (HP).

**Methods:**

A prospective study of 85 patients with suspected HP, of whom 63 were diagnosed with HP due to exposure to avian or fungal antigens. In all cases, EBC samples were collected before and after completion of the SIC and pH values were determined.

**Results:**

Taken as a whole, patients with HP did not present changes in EBC pH after SIC. However, considering only patients with exposure to molds, those diagnosed with HP had a significantly more acid pH post-SIC than those with another diagnosis (*p* = 0.011). This fact is not observed in patients exposed to bird’s antigens. A ROC curve showed that a reduction in EBC pH of 0.3 units or more after SIC in patients diagnosed with HP due to exposure to molds had a sensitivity of 30 % (CI: 12.8 to 54.3 %) and a specificity of 100 % (CI: 65.5 to 100 %).

**Conclusion:**

EBC pH may be useful in interpreting SIC results in patients with HP, especially in those patients exposed to molds. Further studies are now required to test the validity of these proposals.

## Background

Hypersensitivity pneumonitis (HP) is a complex syndrome of variable intensity and clinical history, caused by an immune-mediated inflammation of the lung parenchyma due to the continued inhalation of a wide range of antigens [1]. The increased recognition of exposure to environmental antigens and improvements in the diagnostic tools available have allowed the identification of a variety of environmental and occupational settings that can cause this disease.

HP is difficult to diagnose because of the wide spectrum of clinical variants and the absence of a "gold standard" diagnostic test. In general it is diagnosed on the basis of clinical criteria, among which Schuyler and Cormier’s criteria are the most frequently used [2]. As HP is an immunologically mediated disease, some authors have suggested that the specific inhalation challenge (SIC) could be a useful diagnostic tool [3]. Although the SIC can demonstrate a direct link between exposure to an antigen and impaired lung function in the patient [4, 5], its use in the diagnosis of the HP is limited by the lack of standardization of both the inhalation protocols and the criteria required to define a positive response [6, 7]. Thus, while some authors prioritize the symptoms, others prioritize falls in forced expiratory volume (FVC) and diffusing capacity of the lung for carbon monoxide (DLCO) to establish positivity [3].

In view of the inflammatory nature of most respiratory diseases, the use of non-invasive methods to study lung function for diagnosing or monitoring these pathologies is becoming increasingly common [8]. The study of induced sputum cellularity or of biomarkers in exhaled breath or exhaled breath condensate has proven useful in the evaluation of certain respiratory pathologies [9, 10]. However, the experience with these techniques in patients with HP is limited. Some studies using induced sputum have reported an increase in the total cell count and the lymphocyte count in patients with HP compared to the healthy population [11–13]. Also using induced sputum, our group recently demonstrated that bronchial inflammation is present in patients with HP, and is mainly evidenced by increases in neutrophils that worsen following exposure to the offending antigen during SIC [14]. However, other non-invasive methods for studying pulmonary inflammation have not been analyzed to date in patients with this disease.

The aim of this study was to investigate the influence of SIC on changes of pH values in exhaled breath condensate (EBC) in patients with hypersensitivity pneumonitis (HP).

## Methods

### Study population

**Fig. 1:**
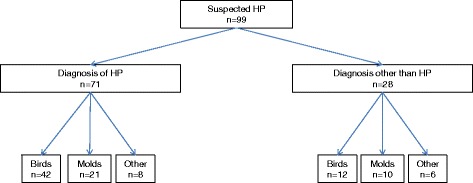
Study sample and agents tested

This prospective, cross-sectional study included all patients older than 18 years, referred to our hospital with suspected HP between 2005 and 2013 and who underwent a SIC (Fig. 1). The study was approved by the local Ethics Committee and all subjects gave informed consent prior to participation (Hospital Vall d’Hebron Ethics Committee approval PS09/01486).

### Diagnostic protocol

Prior to the administration of the SIC, a thorough clinical history was performed along with additional tests: blood count, immunoglobulin G specific for birds and fungi [4, 15], blood calcium levels, angiotensin converting enzyme, lactate dehydrogenase, chest X-ray, high resolution computed tomography, pulmonary function tests including spirometry, static lung volumes and carbon monoxide transfer test, immediate and delayed hypersensitivity skin testing, bronchoscopy with bronchoalveolar lavage and/or transbronchial biopsy and/or criobiopsy. In all cases the diagnosis was made using Schuyler and Cormier’s criteria [2]. None of the patients were taking steroid therapy.

### Lung function studies

All patients underwent forced spirometry, static lung volume study by plethysmography, and determination of the diffusing capacity of the lung for carbon monoxide (DLCO) using the single breath-hold method. These studies were performed on a MasterLab system (MasterLab, Jaegger, Germany) following the joint recommendations of the European Respiratory Society and American Thoracic Society [16] and the Spanish Respiratory Society [17].

### Determination of specific IgG antibodies

Specific IgG to avian sera (pigeon, parrot and parakeet), *Aspergillus fumigatus*, *Penicillium frequentans* and *Mucor mucedo* were measured by an ELISA technique based on the method previously described [4, 18]. Wells of high binding microtiter plates (Costar, Cambridge, MA, USA) were coated with 2 μg protein/well in 0.1 M Na_2_CO_3_/NaHCO_3_ buffer (pH 9.6) at 4 °C overnight. The wells were then washed three times with washing buffer (0.1 M phosphate buffered saline, pH 7.5 containing 0.005 % Tween 20) and blocked with 1 % bovine serum albumin in phosphate buffered saline for 1 h at 37 °C. The specific IgG assays were performed in duplicate by incubating the serum samples at an appropriate dilution for 2 h at 37 °C, and wells were washed four times between each step. A solution of horseradish peroxidase-labeled anti-human IgG monoclonal antibody (clone MH16-1ME, 0.5 mg/mL) diluted at 1:1000 was added to each well and plates were incubated for 2 h at 37 °C. The reaction was developed with 3,3’,5,5’-tetramethylbenzidine (Sigma Chemicals), 3 % H2O2 for 20 min at room temperature in the dark and stopped with 2 M H2SO4. Optical density at 450 nm was measured with a microplate reader (Titertek Multiskan Plus MKII). Results were expressed as absorbance units at 450 nm (A_450 nm_). Values above the mean plus two standard deviations of the results obtained in a control population of 30 healthy individuals previously studied in our laboratory were deemed positive.

### Bronchoscopy techniques: bronchoalveolar lavage (BAL) and transbronchial biopsy (TBB)

Bronchoalveolar lavage was performed according to the recommendations of the European Respiratory Society [19]. The TBB procedure used has been described by other authors [20].

### Antigen extract preparation for specific inhalation challenge

Commercialized extracts (Bial-Aristegui, Bilbao, Spain) from *Penicillium frequentans*, *Aspergillus fumigatus* and *Mucor mucedo* were used to study fungi [18]. The avian sera and pigeon bloom extracts were prepared in our laboratory, as previously described [3, 18].

### Specific inhalation challenge

Informed consent was obtained from all patients prior to performance of SIC. A de Vilbiss 646 nebulizer and Mefar MB3 dosimeter were used, which release the antigenic solution during the first second of each inhalation. The technique consisted of inhaling 2 mL of solution at a 1/100 (0.01 mg/mL) dilution. The patients’ FVC, forced expiratory volume in one second (FEV1), DLCO and temperature were assessed at baseline, at 20 min following exposure, and every hour thereafter for the next eight hours. The SIC was considered positive according to previously published criteria [3, 18]. In patients testing negative on SIC, exposure was repeated 24 h later, at an antigen dilution of 1/10 (0.1 mg/mL). In all cases a baseline test was performed with placebo solution the day before inhalation of the putative causal agent.

### EBC collection

EBC was collected during tidal breathing with a commercially available condenser (EcoScreen; Jaeger, Würzburg, Germany), as described elsewhere [9]. Smokers were advised not to smoke during the 48 h prior to the completion of SIC. Each EBC sample was divided into 500- μL aliquots in two to four plastic tubes. Other aliquots were used to measure the pH before and after deaeration. Baseline EBC pH was recorded 24 h before the SIC and post-SIC EBC pH was recorded 24 h after the last antigenic exposure.

### Measurement of pH in EBC

pH was measured in one of the aliquots immediately after EBC collection and after deaeration with helium (350 mL/min for 10 min), using a calibrated pH meter (Model GLP 21; Crison Instruments SA; Barcelona, Spain) with an accuracy of 0.01 pH, and a probe for small volumes (Crison 50 28; Crison Instruments SA). The probe was calibrated daily with standard pH 7.02 and 4.00 buffers [21].

### Statistical analysis

The Mann–Whitney test and chi-square test were applied to compare continuous and nominal variables, respectively, with a two-sided significance level of 0.05. The consistency of EBC was estimated by evaluating the sensitivity (SE) and specificity (SP) [22] of the method, the positive (PPV) and negative (NPV) predictive values, and the likelihood ratio of a positive (LR+) and negative (LR-) value with 95 % confidence intervals (95 % CI) using the Wilson method [23]. Receiver-operating characteristic (ROC) curves were constructed to determine the cut-off values that best differentiated between having the disease or not [24]. All analyses were done with SPSS, version 17 (Chicago, IL, USA) and, SAS version 9.2 (SAS Institute Inc., Cary, NC, USA).

## Results

A total of 99 patients were studied. Fourteen patients were excluded because the agents suspected of producing HP were neither birds nor molds (Fig. 1). Of the 85 patients studied, 63 were diagnosed with chronic HP, 42 of whom had been exposed to avian proteins and 21 to fungal agents. Of these 63 patients, 52 had a positive SIC. Out of the 52 patients with a positive SIC, 33 were exposed to bird antigens and 19 to molds [3, 18]. After SIC, 20 patients presented a decrease of DLCO >20 %, 15 a decrease of FVC between 10 and 15 % plus an increase >0.5 °C in body temperature, 11 a decrease >15 % in FVC plus a decrease >20 % in DLCO, and finally 6 patients presented a decrease of FVC% >15 %.

Twenty-two patients received diagnoses other than HP, and in all of them the SIC was negative: five were diagnosed with nonspecific interstitial pneumonia, five with sarcoidosis, eight with bronchiectasis, two with non-classifiable pulmonary fibrosis and two with idiopathic pulmonary fibrosis. Of these 22 patients, 12 were exposed to bird’s antigens and ten to fungi.

The clinical characteristics of the study population are presented in Table 1. No significant differences in baseline characteristics were observed according to diagnosis (HP or non-HP) or exposure to bird’s antigens or molds.

**Table 1:** Demographic data and clinical characteristics of the study subjects

	HP			Non-HP		
	*n* = 63			*n* = 22		
	Birds	Molds	*p*	Birds	Molds	*p*
	*n* = 42	*n* = 21		*n* = 12	*n* = 10	
Age, mean (SD), years	57 (11.9)	59 (17.91)	0.789	59 (9,42)	58.33 (14.6)	0.892
Sex, M/F	13/29	8/13	0.8	6/6	5/4	0.449
Smoking (%)						
Smoker	12.8	10.5		11.1	7	
Non-smoker	61.5	52.6		55.6	57	
Ex-smoker	25.6	36.8		33.3	36	
Crackles, *n* (%)	22/35 (62,9)	9/14 (64,3)	0,37	6/8(75)	6/8(75)	0,7
Clubbing, *n* (%)	5/35 (14,3)	0/14 (0)	0,3	2/8(25)	1/8 (12,5)	0,5

**Fig. 2:**
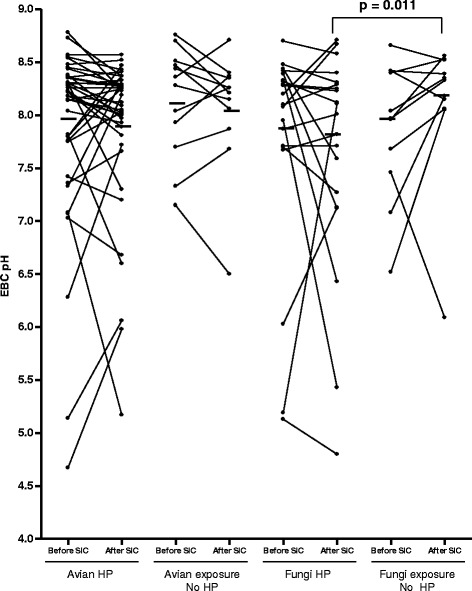
EBC pH before and after SIC in patients diagnosed with HP and in those with diagnoses other than HP

EBC pH values before and after the SIC are displayed in Fig. 2. The mean reduction in EBC pH after SIC in patients with HP was 0.02 in those exposed to bird’s antigens and 0.15 in those exposed to molds; it was not statistically significant in either case (p values 0.903 and 0.634 respectively, Table 2). However, considering only patients with exposure to molds, those diagnosed with HP had a significantly more acid pH post-SIC than those with another diagnosis (*p* = 0.011) (Fig. 2). In fact, in general, post SIC pH was significantly lower in patients diagnosed with HP than in patients with a different diagnosis (*p* = 0.010).

**Table 2:** Immunological, functional pulmonary data, and results from BAL and EBC of the study subjects

	HP			Non-HP		
	*n* = 63			*n* = 22		
	Birds	Molds	*p*	Birds	Molds	*p*
	*n* = 42	*n* = 21		*n* = 12	*n* = 10	
Avian IgG, + / -	22/15	-----	-	5/3	-----	-
Fungal IgG, + / -	-----	11/7	-	-----	2/6	-
FVC %, mean (SD)	74.6 (14.3)	75.53 (12.46)	0.881	75.6 (17.0)	77.33 (12.17)	0.776
DLCO %, mean (SD)	59.5 (17.6)	63.47 (20.33)	0.465	60.3 (15.0)	64.44 (15.89)	0.351
Bronchoscopy (n)	35	17	-	9	9	-
Transbronchial biopsy (n)*	25	13		3	7	
Criobiopsy (n)*	4	0		2	1	
Bronquial biopsy (n)*	0	0		1	0	
Surgical biopsy(n)*	5	0	-	0	0	-
BAL (n)	35	17	-	9	9	-
BAL lymphocytes* %	19.8 (21)	22.41 (17.91)	0.663	20.33 (20.43)	16.1 (11.95)	0.685
SIC, +/−	33/9	19/2	-	0/12	0/10	-
EBC pH before SIC	7.9(0.86)**	7.78(1.03)^***^	0.624	8.14(0.52)	7.92(0.67)	0.245
EBC pH after SIC	7.88(0.74)**	7.63(1.02)^***^	0.270	8.07(0.56)	8.31 (0.19)	0.273

*IgG*, immunoglobulin G; *FVC*, forced vital capacity; *DLCO*: diffusing capacity of the lung for carbon monoxide; *BAL*, bronchoalveolar lavage; *Number of patients in whom the test result was consistent with HP; **p = 0.90; ***p = 0.634

**Fig. 3:**
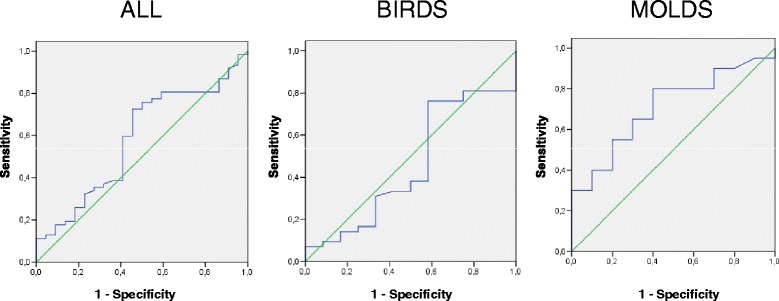
ROC curves: assessment of the most relevant difference in EBC pH after SIC in patients with suspected HP

The sensitivity, specificity and positive and negative predictive values of EBC pH according to the established diagnosis of HP are shown in Table 3. An ROC curve showed that a reduction of EBC pH of 0.3 units or more after SIC had a sensitivity of 25 % (CI: 15.9 to 38.7 %) and a specificity of 81.8 % (CI: 58.9 to 94 %) for the diagnosis of HP in the total study population. Analysing only patients diagnosed with HP due to exposure to fungal antigens, a fall in EBC pH of 0.3 or greater showed a sensitivity of 30 % (CI: 12.8 to 54.3 %) and a specificity of 100 % (CI: 65.5 to 100 %) (Fig. 3). In the group of patients diagnosed with HP due to exposure to avian proteins, a fall in EBC pH of 0.3 or higher showed a sensitivity of 23.8 % (CI: 12.6 to 39.8 %) and a specificity of 66.6 % (35.4 to 88.7 %).

**Table 3:** Exhaled breath condensate diagnostic yield

	All	Birds	Molds
	*n* = 85	*n* = 54	*n* = 31
Sensitivity	25.8	23.8	30
% (CI)	(15.9-38.7)	(12.6-39.8)	(12.8-54.3)
Specificity	81.8	66.6	100
% (CI)	(58.9-94.0)	(35.4-88.7)	(65.5-100)
PPV	80	71.4	100
	(55.7-93.3)	(42.0-90.4)	(51.7-100)
NPV	20	0.2	41.6
	(6.6-44.2)	(0.09-36.1)	(22.8-63.0)
PLR	1.42	0.71	---
	(0.53-3.79)	(0.27-1.87)	
NLR	0.9	1.14	0.7
	(0.77-1.07)	(0.89-1.46)	(0.52-0.93)

Data are expressed as % (95 % CI); *PPV*, positive predictive value; *NPV*, negative predictive value; *PLR*, positive likelihood ratio; *NLR*, negative likelihood ratio

No correlation was observed between the EBC pH before SIC and baseline FVC, DLCO or BAL lymphocytes. Additionally, no correlation was observed between the acidification of pH after SIC and fall in FVC, DLCO or temperature increase.

Considering the false negatives of the SIC, in the group of patients diagnosed of HP due to molds (*n* = 2) one patient experienced a drop in EBC pH > 0.3. In the group of patients diagnosed of HP due to avian proteins (*n* = 9), two patients experienced a drop in EBC pH > 0.3.

## Discussion

The present study demonstrates that a drop in EBC pH of 0.3 or higher after SIC has a sensitivity of 30 % (CI: 12.8 to 54.3 %) and a specificity of 100 % (CI: 65 5–100 %) for diagnosis of HP due to exposure to molds. In the case of HP due to bird proteins, a fall in EBC pH of 0.3 or more after SIC had a sensitivity of 23.8 % (CI: 12.6 to 39.8 %) and a specificity of 66.6 % (35.4 to 88.7 %). This study is the first to analyse the diagnostic performance of the EBC pH in the context of antigen exposure through SIC, in patients with HP due to exposure to bird’s antigens or to molds.

These results may be helpful for establishing a firm diagnosis of HP. In acute forms of the condition the clinical criteria currently in use are sufficient [2], but establishing a firm diagnosis in chronic or subacute forms is much more difficult. It has recently been shown that up to 50 % of cases of idiopathic pulmonary fibrosis may be evolving forms of HP that have not been properly diagnosed [25] and it is in this context where the SIC may be most useful [26].

The SIC has been used to diagnose since the 1960s, but there is still no consensus regarding the variables on which the test’s interpretation should be based. This situation may influence its sensitivity and specificity. In a study with 29 patients basically using clinical criteria such as the onset of symptoms and signs mimicking influenza, Hendrick et al. [5] reported a sensitivity of up to 85 % and a specificity of 95 %. Also basing positivity on clinical symptoms, Ohtani et al. [7] found no false results in a study of 11 patients with bird fancier's lung. However, other authors using criteria based on falls in the values obtained in lung function tests reported sensitivities ranging from 82 to 92 % and specificities between 76 and 100 % [4, 6].

Establishing a positive diagnosis from the appearance of symptoms during the test necessitates a high level of exposure to the antigen, a requirement which may of course have a negative effect on the test’s safety. The use of criteria based on lung function studies to determine diagnosis may allow lower levels of antigen exposure. For example, maximum exposure in Ohtani et al.’s [7] study was 680 μg of avian protein, whereas Morell et al. [4] used a maximum dose of 200 μg of protein. However, this lower antigen exposure during SIC may yield false negatives: evaluating 113 patients, of whom 88 were diagnosed with HP due to different agents, Muñoz et al [26] recently found a sensitivity of 73 % and a specificity of 84 %, and 24 patients finally diagnosed with HP had negative SIC. The results of this study allow us to hypothesize that the measurement of EBC pH during SIC may reduce the number of false negatives and thus improve the test’s diagnostic accuracy. In this regard, three patients in the present study diagnosed with HP but with a negative SIC, experienced a decline of EBC pH greater than 0.3 units after the SIC.

EBC pH measurement is a recently introduced tool which may be useful in assessing various respiratory diseases [27]. Several studies have shown that pH values may fall in non-controlled asthma [28] or in the context of respiratory infections in patients with bronchiectasis, COPD or cystic fibrosis [29]. However there is less experience in the context of interstitial diseases: higher levels have been found in individuals with pulmonary fibrosis [30], and lower levels in patients with asbestosis [31]. EBC pH is the result of a balance between various buffer systems and the production and release of acids and bases in the airways [32]. In healthy individuals, EBC is determined to a significant extent by the NH_4_, HCO_3_ and CO_2_ produced during breathing [33], with the most acidic pH being found in the alveolar lumen in the proximal airway. Inflammatory processes trigger a range of mechanisms which produce acidification of these more proximal airways as a possible innate defense mechanism [34]. These mechanisms are basically the production and excretion of superoxide ions and protons by the respiratory epithelial cells, the inhibition of glutaminase activity in epithelial cells, and finally the recruitment of macrophages and neutrophils whose lysis in the context of inflammation raises the acidity level of the environment [35, 36]. In fact the acidification produced by neutrophil recruitment may well explain some of the findings of the present study. In this sense, previous studies have shown that EBC pH values are decreased during asthma exacerbations but they are not related with spirometric values [37]. Furthermore Kostikas et al [38] demonstrated that EBC pH levels are negatively correlated with the number of eosinophils in sputum and positive correlated with neutrophilic airway inflammation.

Although the presence of lymphocytic inflammation in the alveoli is characteristic in HP, in the bronchi it has been shown that there may be neutrophilic inflammation, especially in cases in which molds are the causative agent [12, 14]. Our group has also recently confirmed that individuals with HP present significantly increased levels of neutrophils in induced sputum following the challenge with fungal agents [14]; this may explain the decrease in pH found in the present study after the challenge test in patients with HP caused by molds. Taken together, these findings also allow us to hypothesize that the mechanism of action of the HP may differ depending on the causative agent.

This study has a number of limitations, some of them deriving from factors that may have influenced the pH values recorded. The most important of these factors is smoking: lower levels of pH in EBC have been reported in healthy individuals exposed to tobacco smoke than in non-exposed subjects [39, 40]. In our study, however, this is unlikely to have been a determinant, as we did not find significant differences in smoking habits in our four study groups. Another limitation is the small number of patients ultimately included. Future studies are needed, with larger numbers of patients, to confirm the sensitivity and specificity of EBC pH for the diagnosis of HP. Moreover, the environmental exposure to specific antigens before SIC is difficult to demonstrate, especially in patients exposed to molds. If experimental exposure to antigen can change pH of EBC, natural exposition can also influence it, so we can not rule out that this environmental exposure might influence the results. Finally, another possible limitation is the fact that EBC was recorded 24 h after the antigen challenge, which may have affected pH levels observed. As yet, the variability of EBC pH after SIC has not been assessed either in occupational asthma or in HP [41]. In any case, the decision to record EBC after 24 h was based on the protocol established by different groups in the context of occupational asthma, in which markers of inflammation are analysed using noninvasive methods in order to help establish the positivity of SIC [42].

## Conclusions

In conclusion, the results of this study suggest that the use of EBC pH may be helpful in the interpretation of SIC in patients with HP. Furthermore, its clinical assessment in the context of this test in relatively straightforward, since it is non-invasive, easy to perform, reproducible and cost-effective. The results of this study also suggest that the use of EBC pH after SIC can reduce the test’s false negative rates. Further studies are now required, with larger numbers of patients, to test the validity of these proposals.

## Abbreviations

CI: Confidence intervals
DLCO: Diffusing capacity of the lung for carbon monoxide
EBC: Exhaled breath condensate
FEV1: Forced expiratory volume in one second
FVC: Forced vital capacity
HP: Hypersensitivity pneumonitis
LR: Likelihood ratio of a negative value
LR: Likelihood ratio of a positive value
NPV: Negative
PPV: Positive
ROC: Receiver-operating characteristic
SE: Sensitivity
SIC: Specific inhalation challenge
SP: Specificity
